# A gap in the recognition of two mycorrhizal factors: new insights into two LysM-type mycorrhizal receptors

**DOI:** 10.3389/fpls.2024.1418699

**Published:** 2024-09-20

**Authors:** Junliang He, Renliang Huang, Xianan Xie

**Affiliations:** ^1^ State Key Laboratory of Conservation and Utilization of Subtropical Agro-Bioresources, Guangdong Laboratory for Lingnan Modern Agriculture, Guangdong Key Laboratory for Innovative Development and Utilization of Forest Plant Germplasm, College of Forestry and Landscape Architecture, South China Agricultural University, Guangzhou, China; ^2^ National Engineering Research Center of Rice, Key Laboratory of Rice Physiology and Genetics of Jiangxi Province, Rice Research Institute, Jiangxi Academy of Agriculture Science, Nanchang, China

**Keywords:** arbuscular mycorrhizal fungi, AM symbiosis, mycorrhizal factors, Myc-LCOs, COs, mycorrhizal biceptor complex, LysM-type mycorrhizal receptors

## Abstract

Arbuscular mycorrhizal (AM) fungi are crucial components of the plant microbiota and can form symbioses with 72% of land plants. Researchers have long known that AM symbioses have dramatic effects on plant performance and also provide multiple ecological services in terrestrial environments. The successful establishment of AM symbioses relies on the host plant recognition of the diffusible mycorrhizal (Myc) factors, lipo-chitooligosaccharides (LCOs) and chitooligosaccharides (COs). Among them, the short-chain COs such as CO4/5 secreted by AM fungi are the major Myc factors in COs. In this review, we summarize current advances, develop the concept of mycorrhizal biceptor complex (double receptor complexes for Myc-LCOs and CO4/5 in the same plant), and provide a perspective on the future development of mycorrhizal receptors. First, we focus on the distinct perception of two Myc factors by different host plant species, highlighting the essential role of Lysin-Motif (LysM)-type mycorrhizal receptors in perceiving them. Second, we propose the underlying molecular mechanisms by which LysM-type mycorrhizal receptors in various plants recognize both the Myc-LCOs and -COs. Finally, we explore future prospects for studies on the biceptor complex (Myc-LCO and -CO receptors) in dicots to facilitate the utilization of them in cereal crops (particularly in modern cultivated rice). In conclusion, our understanding of the precise perception processes during host plant interacting with AM fungi, where LysM-type mycorrhizal receptors act as recruiters, provides the tools to design biotechnological applications addressing agricultural challenges.

## Introduction

Arbuscular mycorrhizal (AM) fungi are crucial components of the plant microbiota (Chialva et al., 2022; Shi et al., 2023). AM symbioses are the beneficial associations established between AM fungi and 72% of land plants (Wang et al., 2017; Genre et al., 2020). AM symbioses have dramatic effects on plant performance and also provide multiple ecological services in natural and agricultural environments, such as plant health, biogeochemical cycling, and soil structure (Smith and Smith, 2012; Choi et al., 2018; Parihar et al., 2020; Weng et al., 2022). The successful establishment of AM symbiosis depends on host plant recognition of the diffusible mycorrhizal (Myc) factors, such as lipo-chitooligosaccharides (LCOs) and chitooligosaccharides (COs) (Maillet et al., 2011; Genre et al., 2013; Feng et al., 2019). Among them, the short-chain COs such as CO4/5 secreted by AM fungi are considered to be the major Myc factors in COs (Genre et al., 2013).

In the past decades, there has been a growing interest among researchers in the identification of Myc factor receptors and their recognition mechanisms, driven by the deciphering of the molecular structures of Myc factors (Maillet et al., 2011; Genre et al., 2013). Previous findings showed a bifunctional LysM receptor Chitin Elicitor Receptor Kinase 1 (CERK1) in rice, encoding *OsCERK1* that regulates both AM symbiosis and Microbe-Associated Molecular Pattern (MAMP)-triggered immune responses (Miyata et al., 2014; Zhang et al., 2015). Moreover, a recent study has shown that *OsCERK1* allelic variation correlates with efficiency of AM fungal colonization and rice growth responses to AM fungi, and rice varieties carrying the *OsCERK1^DY^* (*OsCERK1* from Dongxiang wild rice) had higher symbiotic efficiency with AM fungi and more resistant to pathogen (rice blast) (Huang et al., 2020). Nowadays, this rice variety has been applied to field experiments in Jiangxi province of China. This finding raises the question of whether counter selection for high AM fungal colonization occurred from the origin of plant breeding (Lefebvre, 2020). Correspondingly, it has known that LysM receptors are crucial for orchestrating the AM symbioses, such as the Myc Factor Receptor 1 in rice (OsMYR1) and LysM receptor-like Kinase 10 in tomato (*Solanum lycopersicum*, SlLYK10), which act as the mycorrhizal receptors to recognize Myc-CO4 and Myc-LCOs, respectively (Buendia et al., 2016; Girardin et al., 2019; He et al., 2019).

In this review, we focus on current genetics, biochemical and molecular cell biology, and phylogenetic studies to present the recent advances in our understanding of how host plants recognize the two types of Myc factors during the early phase of AM fungal colonization. Furthermore, we explore the similarities and differences between the recognition mechanisms of two types of Myc factors and LysM receptors in different host plants. Lastly, we outline the potential use of mycorrhizal biceptor complex (double-receptor complexes for Myc-LCOs and CO4/5 in the same plant) as a key tool to design biotechnological applications addressing agricultural challenges.

## Myc-LCOs and -COs elicit the symbiotic signaling in the host plant

“Molecular dialogue” refers to the reciprocal perception process of symbiotic signals between symbiotic partners and encompasses the molecular mechanisms by which plant receptors recognize microbial ligands at the molecular level, and this process is crucial for the symbiotic efficiency of host plants and mycorrhizal-associated microbes (Oldroyd, 2013; Limpens et al., 2015; Xue and Wang, 2020; Wang et al., 2022).

COs are common fungal MAMPs that trigger early Ca^2+^ signaling response in host plants. COs are composed of different numbers of N-acetylglucosamine (GlcNAc) units, and the different lengths of COs can induce different types of plant responses (Mukhtar Ahmed et al., 2020). Previous studies suggest that short-chain COs (mainly CO4 and CO5) secreted from AM fungi activate the common symbiotic signaling pathway (CSSP) and fungal accommodation responses that lead to the establishment of AM symbioses (Genre et al., 2013; Genre and Bonfante, 2020). By contrast, long-chain COs (like CO8) activate the MAMP-triggered immunity and plant defense responses (Shinya et al., 2015; Zhang et al., 2021). However, several recent studies have shown that CO8 can induce nuclear Ca^2+^ spiking in the roots of *Medicago truncatula*, *Lotus japonicus*, and *Oryza sativa* seedlings and activate *M. truncatula* AM symbiotic gene expression (Feng et al., 2019; Zhang et al., 2021; Binci et al., 2024; Fukuda et al., 2024; Zhang et al., 2024). Similarly, CO4 can mildly induce plant immune signal transduction, but its capacity to trigger immune responses is much lower than that of CO8 (Bozsóki et al., 2017; Binci et al., 2024). Therefore, it has been proposed that there may be an overlap in the process of intracellular coding and transducing the two signals after the recognition of CO4/5 and CO7/8 by the host plants, resulting in a similar response to the perception of these two signals by the host plants at the early stage (Giovannetti et al., 2024). Nevertheless, at the later stage, different degrees of polymerization of COs have distinct effects on the colonization levels of AM fungi. Treatment with a mixture of short-chain COs can promote the colonization of AM fungus in the roots of *M. truncatula*, while treatment with CO8 inhibits the colonization of AM fungus in rice roots (Volpe et al., 2020; Zhang et al., 2021). CO8 seems to be unable to consistently induce symbiotic signals in rice from signal recognition to the establishment of AM symbiosis (Zhang et al., 2021). Nonetheless, the impact of CO8 on AM fungal colonization in the roots of diverse plant species remains obscure. Therefore, a more detailed analysis in different plant species with treatments of varying lengths of COs for regulating AM fungal colonization should be conducted in the future.

In addition to Myc-COs, the LCOs, containing four to five GlcNAc units and the side chains modified by lipid chains, were found in AM fungal secretions (Maillet et al., 2011). Like CO4/5, LCOs can elicit AM symbiotic responses in the roots of *M. truncatula*, including triggering nuclear Ca^2+^ spiking and inducing the expression of AM symbiotic genes, consequently promoting AM fungal colonization (Maillet et al., 2011; Sun et al., 2015; Feng et al., 2019). By contrast, some modern rice cultivars only recognize COs and fail to detect LCO signal perception. For example, those cultivated rice seedlings grown under normal nitrogen (N) and phosphate (Pi) supply conditions cannot directly recognize LCOs (Sun et al., 2015; He et al., 2019; Li et al., 2022). Interestingly, a recent study found that AM fungi exhibit varying colonization frequencies in different varieties of indica rice, japonica rice, and wild rice (Huang et al., 2020). Overall, the indica group showed a higher mycorrhizal colonization than that of the japonica group, whereas the wild rice displayed an even higher mycorrhizal colonization than the indica and japonica rice seedlings (Huang et al., 2020). A more recent study showed that the LCO-binding receptor, OsLYK11, is present in the genome of “Xihui18” but is not found in the genome of japonica rice (Ding et al., 2024a). This raises the open question of whether the high colonization selection of AM fungi by different varieties of rice is caused by differential recognition of Myc factors (LCOs or COs).

In addition to symbiotic microorganisms, LCOs have been found in pathogenic microbes and even in some pre-land plant fungal lineages (Rush et al., 2020). Therefore, it has been proposed that LCOs are general signals for fungi rather than symbiosis-specific signals. However, the purified Myc-LCO or Nod-LCO with a similar structure has been shown to induce symbiotic signaling responses in plants and promote AM fungal colonization (Maillet et al., 2011; Feng et al., 2019). Therefore, LCOs secreted by symbiotic microbes at least have the capability to function as the symbiotic signaling molecules. Recently, an interesting study shows that the response of the barley to LCOs is regulated by N and Pi nutritional conditions (Li et al., 2022). The roots of barley seedlings do not exhibit a response to LCOs under normal nutritional conditions. However, under N- and/or Pi-starvation conditions, barley roots activate the perception of LCOs, particularly showing the strongest LCOs signal perception under both N- and Pi-starved conditions (Li et al., 2022).

Interestingly, a previous study showed that LCOs can inhibit the immune responses triggered by immune factors in non-mycorrhizal plants (Liang et al., 2013). Furthermore, the exposure of *M. truncatula* roots to a combination of LCOs and COs has been found to reduce the plant immunity but enhance the symbiotic signaling (Feng et al., 2019), indicating that LCOs and COs signaling synergistically contribute to AM symbiosis. Plant root secretions can stimulate AM fungi to induce the secretion of a variety of Myc-LCOs with different lipid chain modifications and that all of these Myc-LCOs are capable of triggering plant AM symbiosis-related responses (Maillet et al., 2011). Therefore, a hypothesis has been proposed in non-leguminous plants: whether Myc-LCOs secreted by AM fungi can be recognized by both the LYRIIIA receptor and the LYRIA receptor (Wang et al., 2023). Among them, LYRIIIA receptors inhibit the immune responses by recognizing Myc-LCOs, whereas LYRIA receptors activate the symbiotic signaling by recognizing Myc-LCOs (Wang et al., 2023). The processes involved in the recognition of AM symbiotic signals by host plants are diverse and complex. Therefore, we need to discuss the aspect of ligand recognition from the plant LysM receptors in the following text.

## Two plant LysM mycorrhizal receptors recruit two Myc factors

Perception of Myc factors, such as Myc-LCOs and Myc-COs, elicits the cellular and genetic reprograms in the host roots, including the initiation of symbiotic signal transduction, activation of transcription factors, and promotion of functional gene expression (Choi et al., 2018; Shi et al., 2023). Evidence from current studies suggests that a heterodimer LysM receptor complex is required for plants to conduct the perception and transmission of LCO or CO symbiotic signals (Madsen et al., 2011; He et al., 2019; Zhang et al., 2024). Based on the demand to recognize two Myc factors in the same plant species, we define here a concept, “mycorrhizal biceptor complex,” which refers to two different receptor complexes in the same plant that simultaneously recognize and distinguish between Myc-LCO and Myc-CO signals. Certainly, these modules may share the common receptor components, but the recognition of Myc-LCOs and Myc-COs should be distinctly differentiated.

The rice plants require two mycorrhizal LysM receptor-like kinases, OsMYR1 and OsCERK1, for perceiving the Myc-factor CO4 (Zhang et al., 2015; He et al., 2019). It has been revealed that rice OsMYR1, lacking kinase activity in its intracellular domain, exhibits a higher affinity for CO4 compared to the kinase-active OsCERK1 (He et al., 2019). Moreover, CO4 not only facilitates the dimerization of OsMYR1 with OsCERK1 but also induces their phosphorylation for activation of the CSSP (He et al., 2019). It is proposed that OsCERK1 interacts with OsMYR1 in rice as found in the legume *Lotus japonicus* for two LysM-type receptor-like kinases (LjNFR1 and LjNFR5) indispensable for the root nodule symbiosis (Broghammer et al., 2012; Genre and Bonfante, 2020). For example, in leguminous plants such as *M. truncatula*, the CO symbiotic signaling recognition module CERK1-LYR4 and the possible existence of a potential Myc-LCO recognition module LYK3-NFP (LjNFR1-LjNFR5 orthologs) have been identified (Feng et al., 2019). Furthermore, a more recent study further characterizes the symbiotic signal CO recognition module in *M. truncatula* and identified a new member, LYK8, a homolog of the CERK1, which synergistically enhances the recognition of the symbiotic signal COs interacting with CERK1 (Zhang et al., 2024). Additionally, genetic mutations in both *cerk1* and *lyk8* completely block CO signal transduction and AM fungal colonization, indicating that the recognition of the CO symbiotic signal is a key determinant for the successful establishment of AM symbiosis in *M. truncatula* (Zhang et al., 2024). Although LYR4 mediates the symbiotic response to COs, mutations in *LYR4* have no effect on AM symbiosis, indicating the potential existence of alternative LysM receptors that can substitute for its role in CO receptor recognition (Feng et al., 2019). More recently, another new LYK (without kinase activity), namely, MtLYR8, has been shown to bind to CO4, and genetic mutations in *LYR8* reduce the signal transduction of CO4 and the colonization of AM fungus, indicating the involvement of MtLYR8 in the recognition of the symbiotic signal CO4 (Ding et al., 2024a). Taken together, the biochemical and genetic evidence suggests that CERK1/LYK8 may still need other LYKx (without kinase activities) to form multiple dimers (LYR4-CERK1/LYK8-LYKx tetramer) to recognize Myc-COs.

In addition to leguminous plants, solanaceous plants are capable of recognizing LCOs and COs, as evidenced by the identification of the LCO receptor SlLYK10 and the short-chain CO receptor SlLYK9 in *S. lycopersicum* (Girardin et al., 2019; Ding et al., 2024b). Interestingly, the CERK1 receptor undergoes subfunctionalization in *S. lycopersicum*, giving rise to four CERK1-like receptors, namely, SlLYK1, SlLYK11, SlLYK12, and SlLYK13 (Liao et al., 2018). Although SlLYK1 mediates the CO response, knockdown of *SlLYK1*, an ortholog of *MtCERK1*, did not significantly inhibit the AM symbiosis. Instead, only knockdown of *SlLYK12*, an ortholog of *MtLYK8*, significantly suppressed AM fungal colonization, indicating that the recognition mechanism for symbiotic signals LCOs and COs in Solanaceae plants may differ from that in leguminous plants (Liao et al., 2018).

Rice plants can recognize the Myc factor CO4 in the form of an OsCERK1–OsMYR1 complex (He et al., 2019). However, some of the modern domesticated rice roots do not respond to low concentrations of LCOs or trigger nuclear Ca^2+^ spiking under N deficiency (Sun et al., 2015; He et al., 2019). Furthermore, the receptors identified in barley, RLK10 (an ortholog of MtNFP) and RLK2 (an ortholog of MtLYR8), mediate the perception of LCO and CO signals, and genetic mutations in *RLK2* and *RLK10* completely inhibit the recognition of LCOs signals (Li et al., 2022). However, as mentioned above, CERK1 is an indispensable core for Myc factor perception in both dicots and monocots. Therefore, the functionality of CERK1 and its interaction with the RLK2/10 in barley need to be further investigated. Additionally, mutations in *RLK2* and *RLK10* only inhibit the recognition of certain CO4 symbiotic signals, indicating the potential presence of other CO4 recognition modules in barley (Li et al., 2022). Nonetheless, the biceptor complex may also be present in barley and they share RLK2.

Recent experimental evidence suggests that ancient moss plant *Marchantia paleacea* is capable of recognizing LCOs and COs (including CO4 and CO7), indicating that the recognition of symbiotic signals LCOs and COs is not exclusive to angiosperms and can be traced back to even older land plants (Teyssier et al., 2024). In *M. paleacea*, four LysM receptors are present, and severe AM phenotype loss was observed only in the *MpaLYKa* mutant (Teyssier et al., 2024). Given the differences in molecular structure between LCOs and COs, it may be difficult for MpaLYKa to simultaneously recognize and differentiate between LCOs and COs. In addition to MpaLYKa, gene mutations in *MpaLYR* also inhibit the recognition of COs and LCOs signals, indicating the potential role of MpaLYR in the recognition process of COs or LCOs signals, and the recognition of LCOs and COs may be replaced by other non-LysM symbiotic receptors.

Despite the certain progress made so far in the field of research on the plant recognition of single mycorrhizal factor complex, Myc-LCOs or Myc-COs, in a dicot, the molecular mechanisms by which the host plant precisely recognize and discriminate both the Myc-LCOs and Myc-COs derived from an AM fungus still remain unclear. It would be interesting to determine whether CERK1 as the core receptor also interacts with the novel RLKs essential for the Myc-LCO perception in dicots as observed in the rice for OsCERK1 and OsMYR1 heterodimer required for the CO4 recognition.

## From trees to crops: evolutionary history of LysM receptors related to arbuscular mycorrhizae

**Figure 1:**
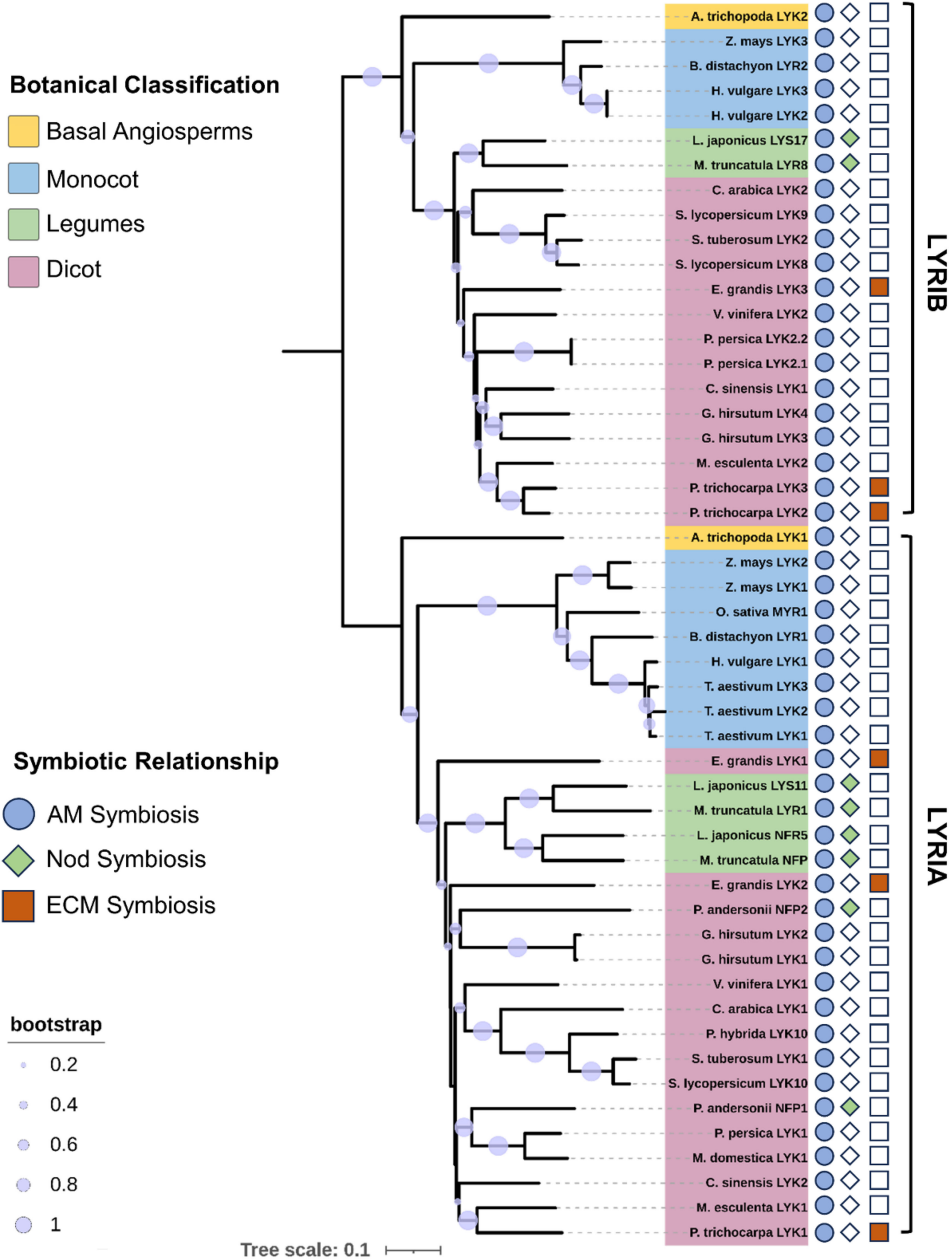
Phylogenetic analysis of mycorrhizal dipeptides in different plants. A phylogenetic tree was constructed for 50 LysM receptors, forming two clades named LYRIA and LYRIB, representing the two evolutionary lineages of the LysM receptor family; A neighbor-joining method was used to construct the phylogenetic tree, which was then expanded 1,000 times and manually edited to remove excess gaps. The resulting tree was visualized using ITOL (Letunic and Bork, 2024).

Based on the current theoretical and experimental evidence, it is becoming increasingly clear that plant LysM receptors play the crucial roles in the Myc-factor perception and AM establishment. Particularly, the CERK1-like receptors with conserved kinase activity act as critical signal transduction receptors, whereas those LysM receptors without kinase activity, such as OsMYR1 and SlLYK10, function as the primary binding receptors for CO4 and LCOs, respectively (Liao et al., 2018; Gibelin-Viala et al., 2019; Girardin et al., 2019; He et al., 2019; Zhang et al., 2019). To further investigate the evolutionary history of mycorrhizal factor receptors in plants, we constructed a rooted phylogenetic tree for LysM-RLKs in LYRI clade (Buendia et al., 2018; Rutten et al., 2020) from various dicots and monocots. Interestingly, we observed the division of the LYRI clade into two groups, LYRIA and LYRIB (**Figure 1**), which is consistent with the previous findings (Buendia et al., 2018; Gough et al., 2018; Ruman and Kawaharada, 2023).

In the LYRIA group, SlLYK10, PhLYK10, and BdLYR1 have been demonstrated to perceive Nod-LCOs and mediate AM symbiotic signaling, whereas legume plants MtNFP and LjNFR5 perceive Nod-LCOs and mediate rhizobia symbiotic signaling (Girardin et al., 2019; He et al., 2019; Ding et al., 2024b). The differentiation of legume model plants, such as *M. truncatula* and *L. japonicus*, which are commonly used to study nodulation, occurred between 50 and 60 million years ago, and many host plant genes associated with root nodule symbiosis underwent duplication events after the Whole Genome Duplication (Young et al., 2011; De Mita et al., 2014), including paralogues of NFP/NFR5, such as LYR1/LYS11 (Young et al., 2011). Despite the fact that the MtLYR1 encoding gene is transcriptionally induced by mycorrhization, and MtLYR1 has the ability to bind LCOs, no defect in AM symbiosis or nodulation phenotype was observed in *mtlyr1* (Cullimore et al., 2023). In contrast to the LysM receptors in the LYRIA group, which have a high affinity to bind LCOs, recent experimental evidence suggests that some of the plant LysM receptor kinases in LYRIB (MtLYR8, BdLYR2, and PhLYK9) have a stronger CO4-binding capacity than Nod-LCOs (Ding et al., 2024a). In addition, the double mutation of *phlyk9* and *phlyk10* resulted in a stronger AM phenotype loss than either single mutation, indicating the importance of biceptor complex in the AM symbiosis (Ding et al., 2024a).

**Figure 2:**
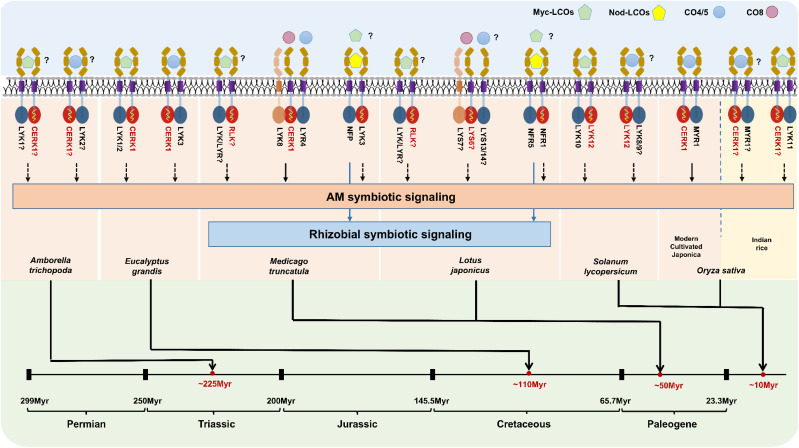
Mycorrhizal biceptor complex in different plants and their underlying molecular regulatory mechanisms. Predicted molecular perception patterns of Myc-LCOs, COs (CO4-CO8), and Nod-LCOs in different mycorrhizal plants. LysM receptors with symbiotic signaling perception capabilities can be divided into two categories: those with kinase activity (red) and those without kinase activity (blue).

Considering that the phenomenon of the mycorrhizal biceptor complex existed in angiosperms, we need to further understand the evolutionary diversity of the mycorrhizal biceptor complex in different plant species. We here discussed the molecular mechanisms of LysM receptor recognition of Myc and/or Nod factors in different plants with differential origins. As shown in **Figure 2**, the objective mycorrhizal plants include the basal angiosperm *Amborella trichopoda*, the woody plant *Eucalyptus grandis*, two model legume plants *M. truncatula* and *L. japonicus*, and one relatively young crop *S. lycopersicum* and domesticated monocot *O. sativa*. Dicotyledonous plants possess a biceptor complex that perceive two mycorrhizal factors, Myc-LCOs and CO4/5 (**Figure 2**). From the phylogenetic tree and the evidence mentioned above, the LysM-RLKs AtrLYK1 and AtrLYK2 were found in the basal angiosperm *A. trichopoda*, belonging to the sister lines of the ancestral LYRIA and LYRIB groups, respectively. Indeed, the AtrCERK1 was also identified in the genome of *A. trichopoda*, indicating that the AtrCERK1/AtrLYK1 dimer and AtrCERK1/AtrLYK2 dimer could recognize the Myc-LCOs and CO4, respectively (**Figure 2**). Similarly, in woody *E. grandis*, there also exist two LysM receptors: the first EgLYK1/2 is in the LYRIA group, whereas the second EgLYK3 is present in the LYRIB group, and the CERK1-like receptor EgCERK1 is also identified from the genome of *E. grandis* (**Figure 1**). Therefore, it is also predicted that EgCERK1/EgLYK1(or EgLYK2) and EgCERK1/EgLYK3 could recognize the Myc-LCOs and CO4/5 from AM fungi. In legume plants, the perception of Nod factors requires MtNFP/LjNFR5 and MtLYK3/LjNFR1, which is similar to either mechanism of Myc-LCOs or CO4/5 perception (Madsen et al., 2011; Pietraszewska-Bogiel et al., 2013; Igolkina et al., 2018). Two recent studies have shown that *M. truncatula* recognizes CO4 and CO8 through the LYK8-CERK1-LYR4 complex and activates AM symbiotic signaling (Feng et al., 2019; Zhang et al., 2024). These studies show that the recognition of COs depends on the active kinase LysM-RLKs from the CERK1 family and a non-active LysM-RLK from LYR4, aligning with studies in different plant species showing that both active kinase and inactive kinase are required for CO perception. In tomato, the identified LCO receptor SlLYK10 may form a complex with SlLYK12 to recognize the Myc-LCOs (Liao et al., 2018; Girardin et al., 2019), whereas SlLYK8/SlLYK9 in the LYRIB group (**Figure 2**) may form a heterodimer with SlLYK12 to recognize the Myc-factor CO4 from AM fungi. In rice, OsMYR1 and OsCERK1 mediate the perception of the mycorrhizal factor CO4 by forming a heterodimer (**Figure 2**). However, modern cultivated Japonica rice seems to have a weak or no perception of LCOs (Sun et al., 2015; He et al., 2019), whereas some cereals (such as barley, wheat, and maize) can recover the perception of LCOs under N and/or Pi starvation (Li et al., 2022). In addition, LCO receptors have been found in other indica species (Ding et al., 2024a), suggesting that some rice varieties may have temporarily inactivated the function of LCO receptors during domestication/breeding and can reactivate their functions under specific conditions such as nutrient starvation. By contrast, in some monocot crops, such as *Zea mays*, *Hordeum vulgare*, and *B. distachyon*, two LysM-RLKs have been found in both the LYRIA and LYRIB groups, indicating that these cereals may be capable of perceiving the Myc-LCOs and CO4 from AM fungi (**Figure 2**). Therefore, it is of great significance to comparatively study the precise perception of Myc-LCOs and CO4/5 between these cereals and rice plants (such as *O. sativa*) in future research. This finding also arises the open question of whether the LCO receptors were retained in the early wild rice.

## Conclusions and perspectives

Myc-LCO and Myc-CO recognition can enhance AM fungal colonization in the dicotyledonous plants, potentially resulting from the crucial role of the mycorrhizal biceptor complex. However, the weak or lack of Myc-LCO perception ability in modern cultivated rice may be a severe limitation for AM fungal colonization, due to inactivation of functional LCO receptors in domesticated rice plants. Therefore, our understanding of the precise perception processes during host plant interacting with AM fungus, where mycorrhizal receptors act as recruiters, provides the tools to design biotechnological applications addressing agricultural challenges. First, an effective approach for enhancing AM fungal colonization in agricultural production is to genetically engineer rice varieties that can perceive both Myc-LCOs and CO4/5 by introducing the optimal Myc-LCO LysM-type receptor allele encoding genes from eudicots. Alternatively, the gene editing technology can be applied to modify the extracellular LysM domains of the potential LysM-type receptor kinases in domesticated rice plants, and ultimately screen for new varieties that can perceive Myc-LCOs and exhibit stronger AM symbiotic benefits. Third, it is highly desirable to identify the efficient Myc-LCO binding receptors in the wild cereals in order to obtain the “inactivated LCO receptors” in the domesticated cereals (such as rice). Except for those prospects, the structural and functional studies are also needed to help to decipher the Myc-LCOs and -CO4/5 binding properties of the mycorrhizal biceptor complex.
